# *P. falciparum* and *P. vivax* Orthologous Coiled-Coil Candidates for a Potential Cross-Protective Vaccine

**DOI:** 10.3389/fimmu.2020.574330

**Published:** 2020-10-21

**Authors:** Imen Ayadi, Saidou Balam, Régine Audran, Jean-Pierre Bikorimana, Issa Nebie, Mahamadou Diakité, Ingrid Felger, Marcel Tanner, François Spertini, Giampietro Corradin, Myriam Arevalo, Socrates Herrera, Valentina Agnolon

**Affiliations:** ^1^Biochemistry Department, University of Lausanne, Epalinges, Switzerland; ^2^University Clinical Research Center (UCRC), University of Sciences, Techniques, and Technologies of Bamako (USTTB), Bamako, Mali; ^3^Department of Internal Medicine II—Nephrology, University Hospital Regensburg, Regensburg, Germany; ^4^Division of Immunology and Allergy, Centre Hospitalier Universitaire Vaudois (CHUV), Lausanne, Switzerland; ^5^Centre National de Recherche et de Formation sur le Paludisme, Ouagadougou, Burkina Faso; ^6^Department of Medical Parasitology and Infection Biology, Swiss Tropical and Public Health Institute, Basel, Switzerland; ^7^Malaria Vaccine and Drug Development Center, Cali, Colombia; ^8^Caucaseco Scientific Research Center, Cali, Colombia

**Keywords:** malaria, *plasmodium falciparum*, *plasmodium vivax*, vaccine, coiled-coil peptides, immune response

## Abstract

Over the last four decades, significant efforts have been invested to develop vaccines against malaria. Although most efforts are focused on the development of *P. falciparum* vaccines, the current availability of the parasite genomes, bioinformatics tools, and high throughput systems for both recombinant and synthetic antigen production have helped to accelerate vaccine development against the *P. vivax* parasite. We have previously *in silico* identified several *P. falciparum* and *P. vivax* proteins containing α-helical coiled-coil motifs that represent novel putative antigens for vaccine development since they are highly immunogenic and have been associated with protection in many *in vitro* functional assays. Here, we selected five pairs of *P. falciparum* and *P. vivax* orthologous peptides to assess their sero-reactivity using plasma samples collected in *P. falciparum-* endemic African countries. *Pf-Pv* cross-reactivity was also investigated. The pairs *Pf27/Pv27, Pf43/Pv43*, and *Pf45/Pv45* resulted to be the most promising candidates for a cross-protective vaccine because they showed a high degree of recognition in direct and competition ELISA assays and cross-reactivity with their respective ortholog. The recognition of *P. vivax* peptides by plasma of *P. falciparum* infected individuals indicates the existence of a high degree of cross-reactivity between these two *Plasmodium* species. The design of longer polypeptides combining these epitopes will allow the assessment of their immunogenicity and protective efficacy in animal models.

## Introduction

Malaria disease was globally estimated at 228 million of cases in 2018 (1). *Plasmodium falciparum* and *P. vivax* are the two most important parasites in terms of infection prevalence and global distribution, and are responsible for more than 98% of global malaria clinical cases. In particular, *P. falciparum* is the most prevalent malaria parasite in Africa, while *P. vivax* is predominant in Asia, Oceania, and America (1).

Although a steady reduction in malaria transmission rates and deaths had been observed during the last two decades as a results of intensified control activities (1), an increase in malaria has been recently reported (2). Additionally, resistance to artemisinin, the main current anti-malarial treatment for *P. falciparum*, has been identified in several countries (3–6), and a reduction of the sensitivity of *P. vivax* parasites to antimalarials like chloroquine and primaquine has been reported (7–10). This represents an enormous challenge to malaria eradication and indicates the need for new control and elimination strategies. In particular, vaccination is considered as a potential cost-effective complement tool for malaria control and elimination. RTS,S/AS01 is the most advanced malaria vaccine candidate to date. It is based on *P. falciparum* circumsporozoite (Pf-CS) protein (11) and has been successfully tested in several Phase III trials in several African countries (12). Other *P. falciparum* candidates based on different approaches have undergone clinical development, such as the PfSPZ vaccine made of irradiated whole sporozoites (13, 14). On the other hand, only a few *P. vivax* vaccine candidates have advanced to Phase I and II vaccine trials (15–18) or are currently under preclinical development (Pvs48) (19). Despite this significant progress, a greater effort has to be invested on the development of malaria vaccines that could target the different parasite phases and species. Considering *P. falciparum* and *P. vivax* global distribution and infection rates, a vaccine providing cross-species protection would strategically reduce the vast majority of malaria clinical cases. The availability of the *Plasmodium* genome and proteome as well as of bioinformatics tools have allowed the identification of parasite proteins containing specific domains with functional importance such as α-helical coiled-coil motifs. Typically, these motifs are short conformationally-stable fragments (around 30/40 residues) composed of two to six α-helices wrapped around each other to form a left-handed supercoil. Each motif is characterized by heptad repeated regions denoted (**a**bc**d**efg)_n_, where “a” and “d” are hydrophobic amino acids whereas the rest are usually hydrophilic residues (20–23). While the hydrophobic residues (**a** and **d**) are crucial for interhelical interactions, the hydrophilic residues are exposed on the surface of the coiled-coil motif and are likely important for interaction with other proteins. Alpha-helical coiled-coil domains can be rapidly produced by chemical synthesis and they auto-fold into their native structure. These peptides are perceived as novel putative antigens for vaccine development (22, 24) and have been investigated for protection against several diseases such as HIV (25), meningitis (26), and influenza (27). In the field of malaria, α-helical coiled-coil peptides were shown to be highly immunogenic in mice (22, 28, 29), with a strong association between antibody levels and clinical immunity (24, 30). Importantly, antibodies specifically directed against these motifs were tested in *ex vivo* biological assays and were shown to induce parasite growth inhibition (22, 29). In detail, α-helical coiled-coil peptides expressed in erythrocytic asexual-stage (22, 23, 28, 29) were *in silico* identified from *P. falciparum* (*n* = 166) and *P. vivax* (*n* = 50) genomes. The corresponding domains were synthesized and tested for their reactivity with sera of individuals from malaria-endemic areas (21, 28, 31).

In the present study, we investigate five α-helical coiled-coil domains that are orthologous between *P. vivax* and *P. falciparum* and share high sequence homology. The five ortholog pairs were selected because they were highly recognized by plasma samples from African malaria-endemic countries. The same panel of orthologs have been previously identified by Cespedes et al. as highly reactive with sera from malaria-endemic areas of Colombia and Papua New Guinea, thus being recognized as potential vaccine candidates (20). These peptides in fact were shown to be highly immunogenic in mice and induce antibodies able to recognize native proteins on *P. vivax* asexual blood stages. We expanded the investigation on the ability of α-helical coiled-coil domains to elicit antibodies with serological cross-reactivity, with the aim of selecting a set of antigenic peptides to be combined in a cross-protective polypeptide antigen for protection against both parasite species.

## Materials and Methods

### Peptides

Alpha-helical coiled-coil *P. falciparum* (*n* = 166) and *P. vivax* (*n* = 50) peptides had been formerly selected from the corresponding malaria proteomes (22, 23, 28). Peptides were chemically synthesized, HPLC purified, and characterized as previously described (22, 28). Based on the data published by Cespedes et al. on the recognition of 50 α-helical coiled coil peptides by human plasma samples, 38 orthologues peptide pairs that showed highest reactivity with plasma from Colombia and Papua New Guinea (PNG) were selected for the present study (28).

### Human Plasma Samples

Plasma samples from Mali were collected in Kenieroba, Bozokin, and Fourda villages located in the Bancoumana town at 75 km from Bamako and in Dangassa village in the Kourouba town at 80 km from Bamako. Plasma were collected from 2009 to 2011 from 35 donors, among which seven were aged from three to 13 years old. Research and ethical clearance for the study was obtained from the Faculty of medicine, pharmacology and odonto-stomatology (FMPOS) of Bamako University (N°0840/FMPOS).

Tanzanian plasma was collected from 37 adult donors of a large-scale community based study undertaken in Ifakara village, Kilombero District, Morogoro Region from 1982 to 1984. Blood samples from adults were taken by finger prick and the serum was kept at −70°C until use. Research and ethical clearance for the study was obtained by the Tanzanian Commission for Science & Technology.

Plasma of eight adult donors from Burkina Faso were collected in the capital city of Ouagadougou. Ethical clearance was obtained from the Ministry of Health, Burkina Faso. After obtaining informed consent from parents and caretakers, heparinized venous blood samples were collected during a cross sectional survey during the malaria low transmission season 1998.

All samples were anonymized and kept at −80°C until use. Samples from Tanzania and Burkina Faso were also used in our previous studies (22, 29, 32). In all studies written informed consent (IC) was obtained from all adult study participants, and an informed assent (IA) was obtained for children through an IC from their parents and legal caretakers. Samples from Burkina Faso were collected during the malaria low transmission season and from donors living in the capital city of Ouagadougou, whereas Tanzanian and Malian samples were collected from residents of the countryside, where people are potentially more exposed to malaria-infected mosquitoes. Plasma samples used as negative controls were collected from Swiss naïve donors who had no history of malaria and no previous travel to malaria-endemic areas.

### ELISA

ELISA was performed using Maxisorp 96-well plates (Thermo scientific, Ref 442404). Plates were coated overnight at 4°C with 5 μg/mL of each peptide. Plates were blocked for 1 h at room temperature (RT) with PBS 1X-milk 3% before being incubated with primary antibodies. Human plasma from Burkina Faso, Tanzania, and Mali donors were tested at the dilution 1:50 or 1:100 in titration two fold dilution series and incubated on plate for 2 h at RT. Goat anti-human IgG conjugated to horseradish peroxidase (HRP) was used as secondary antibody at dilution 1:2,000 (Life technologies, Ref H10307) and 1:1,000 (Invitrogen, Cat No 62-8420) for 1 h at RT. Signal was revealed using TMB substrate reagent (BD OptEIA, cat 555214) for 20 min in the dark at RT, and the reaction was blocked using 1 M sulphuric acid (Merck, 1.00731.1000). Optical density (OD) was measured at 450 nm and 630 nm using a TECAN NanoQuant Infinit M200 PRO spectrophotometer.

### ELISA Competition Assays

In principle, *P. falciparum* or *P. vivax* peptides were adsorbed on the plate as previously described, and then plasma antibodies were incubated, at the dilution giving 50% of the maximal binding to each coated peptide, with a serial dilution of the respective antigen or its orthologue (from 500 μg/mL, 10 fold titration), and without peptide as control (no competition, OD_max_). The resulting inhibited samples were then dispensed onto the coated wells (50 μL/well), and assay was carried on as previously described. Percentage of inhibition was calculated as: 100-(OD−OD_min_)/(OD_max_−OD_min_) ×100, where OD_min_ is the signal in well without serum and OD_max_ is the signal resulting from samples incubated without competitor protein.

### Data Analysis

The percentages of positive responses of serum samples from Mali, Tanzania, and Burkina Faso were evaluated as OD values higher than the mean negative control plus three times the standard deviation at serum dilution 1:200. Orthologue pairs were analyzed in the same experiment on the same day, thus allowing for direct comparison inside each pair. In order to allow for a direct and more informative comparison among peptide pairs, we transformed the OD values in antibody endpoint titers by using an internal positive control. First, OD values were converted to arbitrary units (AU) using the same positive control for each pair of orthologous peptides. The positive control for each couples was a plasma sample giving a similar good response to both orthologues, it was serially diluted and present on each ELISA plate. The 35 plasma Malian samples were individually tested in a twofold dilution series to establish a correlation curve between antibody titers and AU. At this point, antibody titers for all the other field samples were extrapolated from the correlation curve log (titer) = f [log(AU)], with a coefficient of variability between expected and extrapolated titers below 25%. For both *P. falciparum* and *P. vivax* peptides, titers were extrapolated from the same correlation curve in order to directly compare both orthologues. Friedman test followed by Dunn's multiple comparison test were performed for a collective analysis of titers from the three countries against all peptides.

In cross-reactivity experiments, samples were considered cross-reactive if positive to both orthologues. The percentage of cross-reactivity was calculated over the total of samples positive for at least one of the two orthologues.

## Results and Discussion

### *P. falciparum* and *P. vivax* α-Helical Coiled-Coil Peptides Are Similarly Recognized by Plasma From *P. falciparum* Endemic Regions

Previous work published by Cespedes et al. reported the recognition of 50 α-helical coiled coil peptides by human plasma samples from Colombia and Papua New Guinea (PNG), identifying 38 peptides that showed the highest sero-reactivity (28). In the present study we expanded the analysis of the 38 peptides and their orthologous by testing them with three panels of plasma samples obtained from African donors living in Mali, Tanzania or Burkina Faso (Supplementary Table 1). Plasma samples were collected during the ‘90s and the first decade of the current century, when these countries were endemic for *P. falciparum* with no *P. vivax* detection (33, 34). Indeed, low prevalence of *P. vivax* was only recently reported in Mali (35, 36). From the panel of 38 *P. falciparum* and *P. vivax* orthologues, five were highly recognized by plasma samples from African malaria-endemic countries and were selected for further studies (Table 1). Notably, these five pairs coincided with the *P. vivax* peptides identified by Cespedes et al. (28) as potential vaccine candidates. The *P. falciparum* orthologues *Pf* 27, *Pf* 43, *Pf* 45, *Pf* 82.02, and *Pf* 96.03 were the only peptides recognized by at least 90% of the tested samples, with their *P. vivax* orthologues being recognized by 69 to 98% of the samples (Supplementary Table 1).

**Table 1:** Homology between *P. falciparum* and *P. vivax* peptides.

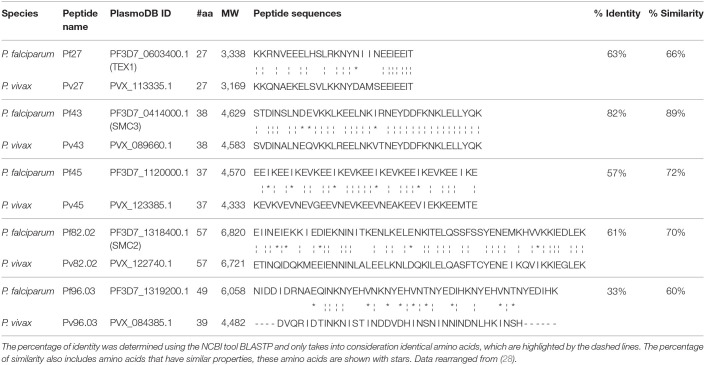

The selected five pairs of orthologous peptides have lengths between 27 and 59 amino acids and display sequence identities ranging from 33 to 82% and a similarity higher than 60% (Table 1). Orthologues *Pf* 27/*Pv*27, *Pf* 43/*Pv*43, *Pf* 45/Pv45, and *Pf* 82.02/*Pv*82.02 share more than 50% identity (82, 63, 61, and 57%, respectively), while the couple *Pf* 96.03/*Pv*96.03 have the lowest identity percentage (33%). However, this low Pf/Pv identity appears to be balanced by a 60% similarity that results in 100% recognition of the *P. falciparum* and 98% of the *P. vivax* orthologues by the African sample panel (Supplementary Table 1), underlining the importance of charged amino acids for antigen-antibody binding.

The antigenicity of the orthologous coiled-coil peptides was analyzed with the panel of plasma from African donors, and median OD values for each peptide together with percentages of positive responses are summarized in Table 2. No significant difference was observed among the median OD of *P. falciparum* peptides and their *P. vivax* orthologue. A complete descriptive analysis showing direct comparison between the recognition of *P. falciparum* vs. *P. vivax* coiled-coil peptides and each plasma in the African donors panel is provided in Supplementary Figure 1. Globally, from Table 2 and Supplementary Figure 1 we can recognize the orthologues pair *Pf* 45/*Pv*45 as the one resulting in the highest OD values. As an additional observation, the recognition of *P. vivax* peptides by plasma samples from *P. falciparum* endemic regions is an indication of cross-reactivity between the two *Plasmodium* species that could be exploited for the generation of a cross-protective vaccine. This type of analysis could now be expanded to *P. ovale* and *P. malariae* that are prevalent in most of the regions where malaria in endemic. However, their genomes have only been available in the last 2 years, but not at the time of peptide selection and synthesis (37).

**Table 2:** *Pf* and *Pv* orthologous peptides are both recognized by African samples.

**Peptide name**	**Mali** ***n*** **=** **35**		**Tanzania** ***n*** **=** **37**		**Burkina faso** ***n*** **=** **8**		**Total** ***n*** **=** **98**		**Switzerland** ***n*** **=** **6**	
	**Median OD**	**% positivity**	**Median OD**	**% positivity**	**Median OD**	**% positivity**	**Median OD**	**% positivity**	**Median OD**	**Cut-off**
*Pf27*	0.140 (0.070;0.287)	89	0.161 (0.072;0.302)	95	0.084 (0.053;0.167)	75	0.139	90	0.015 (0.010;0.024)	0.056
*Pv27*	0.136 (0.055;0.328)	97	0.181 (0.083;0.331)	97	0.070 (0.056;0.147)	88	0.137	96	0.020 (0.019;0.020)	0.034
*Pf43*	0.039 (0.026;0.073)	91	0.145 (0.069;0.219)	100	0.051 (0.032;0.103)	100	0.071	96	0.008 (0.007;0.009)	0.018
*Pv43*	0.053 (0.032;0.102)	80	0.089 (0.059;0.176)	97	0.080 (0.032;0.097)	100	0.072	90	0.014 (0.011;0.015)	0.026
*Pf45*	0.160 (0.080;0.410)	97	0.397 (0.182;0.912)	100	0.215 (0.050;0.514)	75	0.289	96	0.011 (0.009;0.018)	0.037
*Pv45*	0.280 (0.095;0.563)	69	0.265 (0.106;0.665)	68	0.314 (0.156;0.328)	75	0.282	69	0.009 (0.007;0.050)	0.128
*Pf82.02*	0.114 (0.074;0.209)	91	0.290 (0.150;0.457)	100	0.125 (0.044;0.307)	88	0.163	95	0.010 (0.007;0.012)	0.033
*Pv82.02*	0.094 (0.056;0.158)	83	0.219 (0.148;0.407)	100	0.139 (0.063;0.178)	100	0.151	93	0.016 (0.009;0.025)	0.052
*Pf96.03*	0.131 (0.105;0.274)	100	0.269 (0.140;0.597)	100	0.070 (0.062;0.169)	100	0.180	100	0.015 (0.013;0.018)	0.025
*Pv96.03*	0.150 (0.063;0.255)	97	0.294 (0.168;0.468)	100	0.070 (0.054;0.140)	88	0.175	98	0.011 (0.007;0.018)	0.031

*Median OD calculated at sample dilution 1:200 and the respective interquartile ranges (in parenthesis) are indicated. The percentage of positive responses is calculated as the percentage of OD values higher than the mean OD plus three times the standard deviation of the negative control at the dilution 1:200 (indicated as cut-off)*.

**Figure 1:**
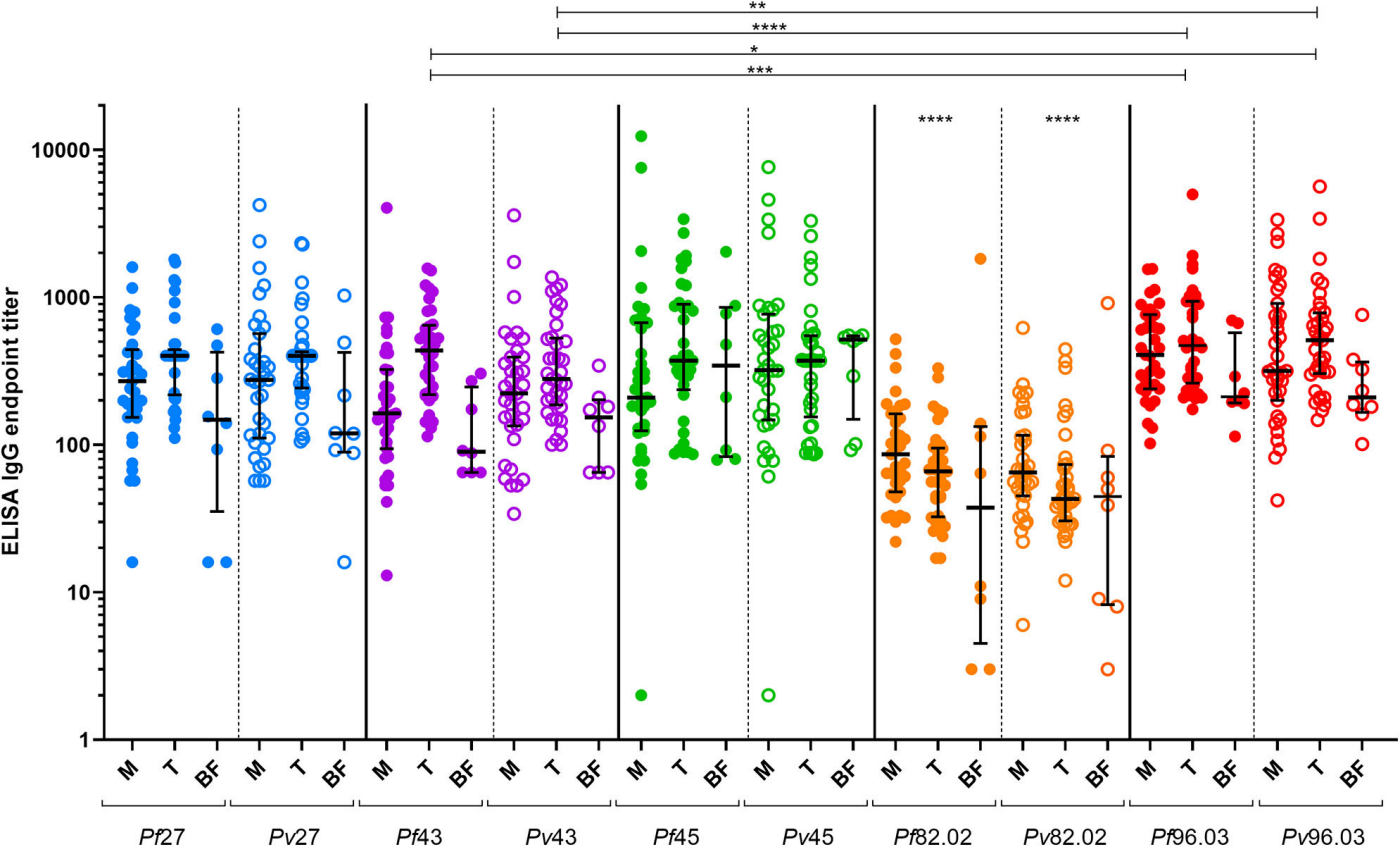
Antibody response to *P. falciparum* and *P. vivax* orthologous peptides. The dot-plot graph shows IgG endpoint titers to the different pairs of orthologous peptides tested with samples from Mali (M, *n* = 35), Tanzania (T, *n* = 37), and Burkina Faso (BF, *n* = 8). Median and interquartile ranges (25th and 75th percentiles) are shown. Median titers by peptide considering the three countries collectively: *Pf27* = 335; *Pv27* = 354; *Pf43* = 237; *Pv43* = 246; *Pf45* = 315; *Pv45* = 371; *Pf82.02* = 68; *Pv82.02* = 54; *Pf96.03* = 429; *Pv96.03* = 371. Friedman test followed by Dunn's multiple comparison test were performed. Statistical significance represented in the graph is referred to a collective analysis of titers from the three countries.

**Figure 2:**
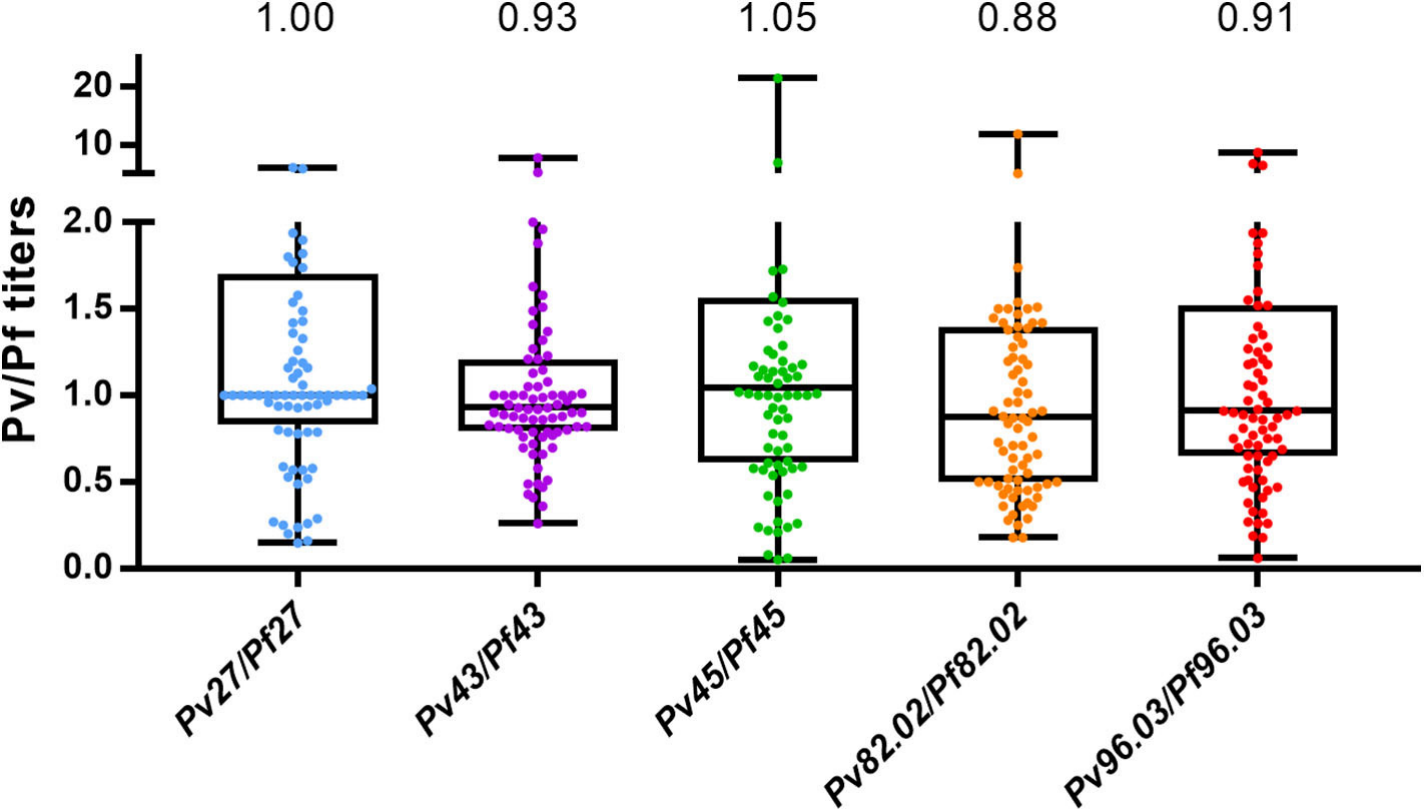
*P. falciparum* and *P. vivax* orthologous peptides are similarly recognized. The graph shows the titer ratio between *Pf* and *Pv* orthologues. Peptides were tested with samples from Mali (*n* = 35), Tanzania (*n* = 37), and Burkina Faso (*n* = 8) and results of the three countries are combined. Median and interquartile range (25th and 75th percentiles) are shown. Values the median. One dot corresponds to one sample. Kruskal Wallis followed by Dunn's multiple comparison test: no significant differences.

Antibody response to the five pairs of orthologous peptides (Figure 1) showed no statistical difference between *P. falciparum* vs. *P. vivax* titers when data from each individual country were analyzed. However, we could identify a particular trend in plasma from Burkina Faso showing the lowest titers probably because they were collected during the low transmission season and from donors living in the capital that are potentially less exposed to *Plasmodium*-infected mosquitoes. Similarly, plasma from Mali showed generally lower titers than plasma from Tanzania. Indeed, the former included children and adolescents among the donors panel, which have statistically had fewer contacts with *Plasmodium*-infected mosquitos. No significant difference was observed between *Pf* and *Pv* titers when all ELISA results from the three countries were combined. These results strongly suggest a cross-reactivity between *P. falciparum* and *P. vivax* coiled-coil peptides. As described by Olugbile et al. (29), the synthesis of a candidate vaccine consisting of two or more polypeptides will permit to maximize the proportion of the population that will respond to such a candidate while conserving the individual functional capacities of each constituent peptide. With reference to our data, we found titers of peptide 82.02 from both *Plasmodium* species to be significantly lower than those of the other peptides (Figure 1), identifying this pair as not appealing to be included in the combined polypeptide vaccine. In addition, the peptides pair 82.02 showed the biggest difference between the titers of the *P. falciparum* or *P. vivax* peptide (Figure 2). Concerning the other pairs, antibody titers resulted to be similar between each ortholog, and the ratio between *P. falciparum* and *P. vivax* peptide titers was close to one (Figure 2), suggesting a good cross-reactivity that might contribute to cross-species clinical protection.

### Cross-Reactivity Between *P. falciparum* and *P. vivax* α-Helical Coiled-Coil Peptides

To assess the frequency of plasma samples recognizing both orthologous peptides, we introduced a definition of positivity stricter than that applied before and only plasma samples that were positive for at least one of the two orthologues and had a titer higher than 200 were considered. Pairs *Pf* 27/*Pv*27, *Pf* 43/*Pv*43, and *Pv*96.03/*Pv*96.03 were recognized in more than 60% of the samples (62, 71, and 78%, respectively) (Table 3). Surprisingly, recognition of both peptides of pairs *Pf* 45/*Pv*45 and *Pf* 82.02/*Pv*82.02 was <50%, even though the similarity percentage between these two pairs of orthologues is around 70% and thus higher than that of the more cross-reactive pairs *Pf* 27/*Pv*27 and *Pv*96.03/*Pv*96.03, as shown in Table 1.

**Table 3:** Frequency of donors recognizing both *Pf* and *Pv* orthologous peptides.

**Orthologous peptides**	***Pf27/Pv27***	***Pf43/Pv43***	***Pf45/Pv45***	***Pf82.02/Pv82.02***	***Pf96.03/Pv96.03***
Number of samples positive to at least one orthologue	65	55	65	11	73
% Cross-reacting samples	62% (40/65)	71% (39/55)	46% (30/65)	36% (4/11)	78% (57/73)

*Samples from the three African countries were considered cross-reactive when positive to both orthologues. The percentage of cross-reactivity was calculated over the total of samples positive for at least one of the two orthologues*.

To experimentally analyse cross-reactivity between *P. falciparum* and *P. vivax* orthologous peptides, competition ELISA were performed on each peptide. Competitors were the peptide itself or its ortholog. In Tables 4A,B (left panels), we reported the results of competition ELISA for a small panel of plasma for each peptide as representative of all the others. We identified few cases of cross-reactive inhibition, for example plasma A005 and KA006 were almost completely inhibited by *Pf* 27 and *Pv*27, resulting in a percentage of inhibition higher than 80%, the coated peptide being the same as the competitor or its ortholog. Overall, the results of competition ELISA were diverse, ranging from complete to zero inhibition, irrespective if the peptide used as inhibitor was the same as the coated one or its orthologue. We initially interpreted this as an effect of the lower antibody-antigen affinity in solution compared to the avidity of antibodies for an antigen adsorbed to the plate surface, which, together with their three-dimensional coiled-coil structure, is favored by the improved proximity among them. To investigate this possibility, we tested if the recognition among the panel of African plasma and the peptides was really specific. When testing each plasma sample without coating or in presence of the coiled coil peptides, some plasma gave a background signal equal or higher than the actual antigen-antibody reaction (Tables 4A,B, right panels).

**Table 4:** Percentages of inhibition obtained in competition ELISAs.

**% Competition in ELISA**				**Direct ELISA (OD)**	
**(A)**					
**Coating**	**Pf27**			**Non-coated**	**Pf27**
**Inhibitor**	**Pv27**	**Pf27**			
A005	82.2	92.2	*	0.067	0.374
KA006	84.8	87.3	*	0.210	0.936
NI MK	24.6	67.4		0.218	0.726
BFO90	53.3	61.72		0.126	0.365
**Coating**	**Pf43**			**Non-coated**	**Pf43**
**Inhibitor**	**Pv43**	**Pf43**			
F28	1.4	0		0.213	0.203
F34	6.2	10.6		0.799	0.794
A025	38.3	5.8		0.698	1.257
KA007	0	0		1.082	1.194
**Coating**	**Pf45**			**Non-coated**	**Pf45**
**Inhibitor**	**Pv45**	**Pf45**			
BFO70	0	88		0.111	0.986
BFO80	0	93.9		0.059	1.451
KA005	0	80.2		0.114	1.791
Adult3	24.2	62.3		0.071	1.050
**Coating**	**Pf82.02**			**Non-coated**	**Pf82.02**
**Inhibitor**	**Pv82.02**	**Pf82.02**			
BFO40	87.4	94.6	*	0.029	0.482
F15	0	0		0.582	0.527
KA005	0	21.1		0.509	0.977
KA007	0	0		0.835	0.866
**Coating**	**Pf96.03**			**Non-coated**	**Pf96.03**
**Inhibitor**	**Pv96.03**	**Pf96.03**			
A005	0	58.6		0.164	0.302
A024	0	4.1		0.777	0.990
KA007	0	0		0.874	0.751
KN0852	0	85.3		0.107	0.633
**(B)**					
**Coating**	**Pv27**			**Non-coated**	**Pv27**
**Inhibitor**	**Pv27**	**Pf27**			
A005	92.1	91.8	*	0.101	0.723
KA006	90.2	87.5	*	0.215	1.116
NI MK	45.5	36.2		0.321	0.809
Adult3	30.1	49		0.267	0.596
**Coating**	**Pv43**			**Non-coated**	**Pv43**
**Inhibitor**	**Pv43**	**Pf43**			
A025	79.7	66.7	*	0.224	0.750
F8	0	0		0.761	0.761
F15	17.6	2.9		1.156	1.140
F22	29.1	10.1		0.453	0.560
**Coating**	**Pv45**			**Non-coated**	**Pv45**
**Inhibitor**	**Pv45**	**Pf45**			
F10	90.6	77.1	*	0.050	0.221
F14	90.2	17.9		0.128	0.572
F25	51.8	21.6		0.110	1.129
F26	6.5	22		0.098	1.658
**Coating**	**Pv82.02**			**Non-coated**	**Pv82.02**
**Inhibitor**	**Pv82.02**	**Pf82.02**			
BFO40	82.4	90.2	*	0.026	0.401
F15	5.1	0		0.828	0.919
KA005	50.1	6.2		0.500	1.017
KA007	12.2	30.8		0.913	1.128
**Coating**	**Pv96.03**			**Non-coated**	**Pv96.03**
**Inhibitor**	**Pv96.03**	**Pf96.03**			
Adult3	85.2	0		0.148	0.897
F6	77.1	0.5		0.104	0.868
F24	39.9	0.1		0.237	0.290
F28	5.9	0		0.222	0.203

Percentages of inhibition obtained in competition ELISAs with P. falciparum (A) or P. vivax (B) coated plates and in presence of the two respective orthologues as inhibitors. A direct ELISA with peptide-coated or non-coated wells was performed in parallel in order to allow for a correct interpretation of the competition ELISA results. Samples were considered positive when the peptide-specific OD was at least three times higher than its OD in the non-coated wells. Values in bold green are positive and representative of an effective and acceptable inhibition. Cross-reactive inhibitions are highlighted with a*. Positivity was rejected in 13 out of 40 tested samples (in red).

This phenomenon has been already reported in the literature (38–41) and the term serum-specific background noise (SSBN) has been coined for this observation known to affect 4–32% of sera, especially when tested individuals had recent or ongoing bacterial infections at the time of blood sampling. In presence of SSBN, subtraction of the aspecific signal from the antigen-specific signal is suggested before data analysis, in order to increase assay sensitivity (38, 40). In the present investigation we decided to apply a more strict rule. Each plasma sample was tested without coating or in the presence of the coiled coil peptides, and was considered positive when its peptide-specific OD value was at least three times its OD value in the non-coated wells (Tables 4A,B, right panels). As a result, positivity was rejected in 13 out of 40 tested samples (highlighted in red in Table 4), corresponding to a 32.5% of samples with aspecific reactivity. Frequently, samples with background noise were associated to absence of inhibition with both orthologues. This is for example the case for plasma KA007, which gave similar high background with different peptides, confirming this effect is indeed sample-specific rather than due to the coating. Given these results, we wish to highlight their importance in order to raise awareness of SSBN in the scientific community. This issue should be taken into consideration—as in the present study—for proper interpretation of ELISA results. Indeed, SSBN analysis led us to identify real inhibition in almost half (19/40) of the samples tested (highlighted in bold and green in Table 4).

Particularly, *Pf27/Pv27* and *Pf45/Pv45* showed clear cross reactivity with the potential to be selected as candidates. Interestingly, these peptides have been previously tested for their antigenicity in sera obtained from donors from both *P. falciparum* and *P. vivax* endemic countries, and purified specific IgG antibodies showed capacity to recognize native proteins in IFAT and functional activity in ADCI assays (22, 28, 29). In addition, association of antibody responses with protection against infection from both parasites was observed (24, 29). Such results support the selection of *Pf27/Pv27* and *Pf45/Pv45* as promising candidates to be combined in a polypeptide vaccine conferring cross-protection against *P. falciparum* and *P. vivax*. The combination of two or eventually more antigens as a single product is cost-effective in terms of vaccine manufacturing and easy to scale-up.

## Conclusions

We showed the recognition of *P. vivax* peptides by plasma of *P. falciparum* infected people living in Mali, Tanzania, and Burkina Faso, thus establishing the existence of a high degree of cross-reactivity between the ortholog coiled-coil domains of the two *Plasmodium* species. Current studies are focused on the development of hybrid nanoparticles, as recently described by Karch et al. (42), containing *P. falciparum* and *P. vivax* cross-reactive coiled-coil domains capable of eliciting a protective response toward the two parasites in experimental animals.

## Data Availability Statement

The raw data supporting the conclusions of this article will be made available by the authors, without undue reservation.

## Ethics Statement

The studies involving human participants were reviewed and approved by Faculty of medicine, pharmacology and odonto-stomatology (FMPOS) of Bamako University (N°0840/FMPOS); Tanzanian Commission for Science & Technology; Ministry of Health, Burkina Faso. Written informed consent to participate in this study was provided by the participants' legal guardian/next of kin.

## Author Contributions

GC, MA, and SH: conceived the study. IA, J-PB, and VA: performed the experiments. IA, RA, and VA: analyzed the data. SB, IN, MD, IF, and MT: contributed with reagents and material. IA: writing-original draft. VA, RA, IF, FS, and GC: writing-review and editing. All authors discussed the results, contributed to the revision of the manuscript, and approved the final version.

## Conflict of Interest

The authors declare that the research was conducted in the absence of any commercial or financial relationships that could be construed as a potential conflict of interest. The reviewer TT declared a past co-authorship with one of the author MD to the handling editor.
