# Supplementary material for: P. falciparum and P. vivax Orthologous Coiled-Coil Candidates for a Potential Cross-Protective Vaccine
Source: Front Immunol. 2020 Oct 21;11:574330. doi: 10.3389/fimmu.2020.574330 (PMC7609509; doi:10.3389/fimmu.2020.574330)

**Supplementary materials**

**Table S1 – Percentage of positive response against *P. falciparum* and *P. vivax* peptides**

Peptides were tested with samples from Mali, Tanzania, and Burkina Faso. The percentages of positive responses were evaluated as OD values higher than the mean negative control plus 3 times the standard deviation at serum dilution 1:200. Nd stands for not done, samples not analysed because of scarcity of material. Five pairs of orthologues peptides (highlighted in grey) were selected for further studies because of their highest percentages of positivity.

| ***P. falciparum*** | | | | |  | ***P. vivax*** | | | | |
| --- | --- | --- | --- | --- | --- | --- | --- | --- | --- | --- |
| **% of positive samples** | | | | |  | **% of positive samples** | | | | |
|  | **Mali**  **n=35** | **Tanzania**  **n=37** | **Burkina Faso**  **n=8** | **Total**  **n=80** |  |  | **Mali**  **n=35** | **Tanzania**  **n=37** | **Burkina Faso**  **n=8** | **Total**  **n=80** |
| ***Pf5*** | nd | 84 | nd | 84 |  | ***Pv5*** | nd | 92 | nd | 92 |
| ***Pf12*** | 60 | 78 | 38 | 66 |  | ***Pv12*** | 23 | 59 | 0 | 38 |
| ***Pf27*** | 89 | 95 | 75 | 90 |  | ***Pv27*** | 97 | 97 | 88 | 96 |
| ***Pf39*** | nd | 49 | nd | 49 |  | ***Pv39*** | nd | 46 | nd | 46 |
| ***Pf42*** | 40 | 35 | 38 | 38 |  | ***Pv42*** | 43 | 32 | 50 | 39 |
| ***Pf43*** | 91 | 100 | 100 | 96 |  | ***Pv43*** | 80 | 97 | 100 | 90 |
| ***Pf45*** | 97 | 100 | 75 | 96 |  | ***Pv45*** | 69 | 68 | 75 | 69 |
| ***Pf52*** | 69 | 73 | 63 | 70 |  | ***Pv52*** | 23 | 65 | 0 | 40 |
| ***Pf53*** | nd | 27 | nd | 27 |  | ***Pv53*** | nd | 30 | nd | 30 |
| ***Pf55*** | 66 | 46 | 88 | 59 |  | ***Pv55*** | 26 | 22 | 63 | 28 |
| ***Pf60*** | 17 | 8 | 25 | 14 |  | ***Pv60*** | 100 | 59 | 100 | 81 |
| ***Pf63*** | nd | 57 | nd | 57 |  | ***Pv63*** | nd | 100 | nd | 100 |
| ***Pf73*** | 6 | 11 | 0 | 8 |  | ***Pv73*** | 86 | 73 | 88 | 80 |
| ***Pf77*** | 77 | 84 | 63 | 79 |  | ***Pv77*** | 34 | 57 | 0 | 41 |
| ***Pf82.02*** | 91 | 100 | 88 | 95 |  | ***Pv82.02*** | 83 | 100 | 100 | 93 |
| ***Pf83*** | 31 | 38 | 0 | 31 |  | ***Pv83*** | 63 | 51 | 13 | 53 |
| ***Pf87*** | nd | 32 | nd | 32 |  | ***Pv87*** | nd | 27 | nd | 27 |
| ***Pf88*** | nd | 35 | nd | 35 |  | ***Pv88*** | nd | 24 | nd | 24 |
| ***Pf89*** | 57 | 19 | 38 | 38 |  | ***Pv89*** | 97 | 16 | 75 | 58 |
| ***Pf90*** | nd | 62 | nd | 62 |  | ***Pv90*** | nd | 73 | nd | 73 |
| ***Pf91*** | 43 | 57 | 13 | 46 |  | ***Pv91*** | 51 | 89 | 25 | 66 |
| ***Pf92*** | 40 | 73 | 13 | 53 |  | ***Pv92*** | 69 | 86 | 38 | 74 |
| ***Pf95*** | 43 | 57 | 25 | 48 |  | ***Pv95*** | 20 | 41 | 13 | 29 |
| ***Pf96.03*** | 100 | 100 | 100 | 100 |  | ***Pv96.03*** | 97 | 100 | 88 | 98 |
| ***Pf97.02*** | 20 | 41 | 50 | 33 |  | ***Pv97.02*** | 74 | 35 | 50 | 54 |
| ***Pf100*** | 57 | 76 | 50 | 65 |  | ***Pv100*** | 37 | 49 | 38 | 43 |
| ***Pf102*** | 6 | 30 | 0 | 16 |  | ***Pv102*** | 3 | 54 | 13 | 28 |
| ***Pf104.1*** | nd | 73 | nd | 73 |  | ***Pv104.1*** | nd | 38 | nd | 38 |
| ***Pf106*** | 23 | 70 | 0 | 43 |  | ***Pv106*** | 57 | 59 | 13 | 54 |
| ***Pf107*** | nd | 35 | nd | 35 |  | ***Pv107*** | nd | 76 | nd | 76 |
| ***Pf110*** | nd | 38 | nd | 38 |  | ***Pv110*** | nd | 32 | nd | 32 |
| ***Pf112*** | 31 | 68 | 13 | 46 |  | ***Pv112*** | 51 | 70 | 75 | 63 |
| ***Pf121*** | 6 | 43 | 0 | 23 |  | ***Pv121*** | 69 | 100 | 63 | 83 |
| ***Pf123*** | 40 | 54 | 25 | 45 |  | ***Pv123*** | 69 | 81 | 50 | 73 |
| ***Pf125*** | nd | 19 | nd | 19 |  | ***Pv125*** | nd | 32 | nd | 32 |
| ***Pf127*** | nd | 38 | nd | 38 |  | ***Pv127*** | nd | 78 | nd | 78 |
| ***Pf131*** | nd | 32 | nd | 32 |  | ***Pv131*** | nd | 68 | nd | 68 |
| ***Pf145*** | 71 | 89 | 100 | 83 |  | ***Pv145*** | 74 | 92 | 100 | 85 |

**Figure S1 -** **Antibody response to *P.falciparum* and *P.vivax* orthologous peptides.** The histograms show antibody titers to the different couples of orthologous peptides: (A) *Pf*27/*Pv*27, (B) *Pf*43/*Pv*43, (C) *Pf*45/Pv45, (D) *Pf*82.02/*Pv*82.02, (E) *Pf*96.03/*Pv*96.03. Peptides were tested with samples from Mali (n=35), Tanzania (n=37), and Burkina Faso (n=8). Mean and standard deviation are shown.


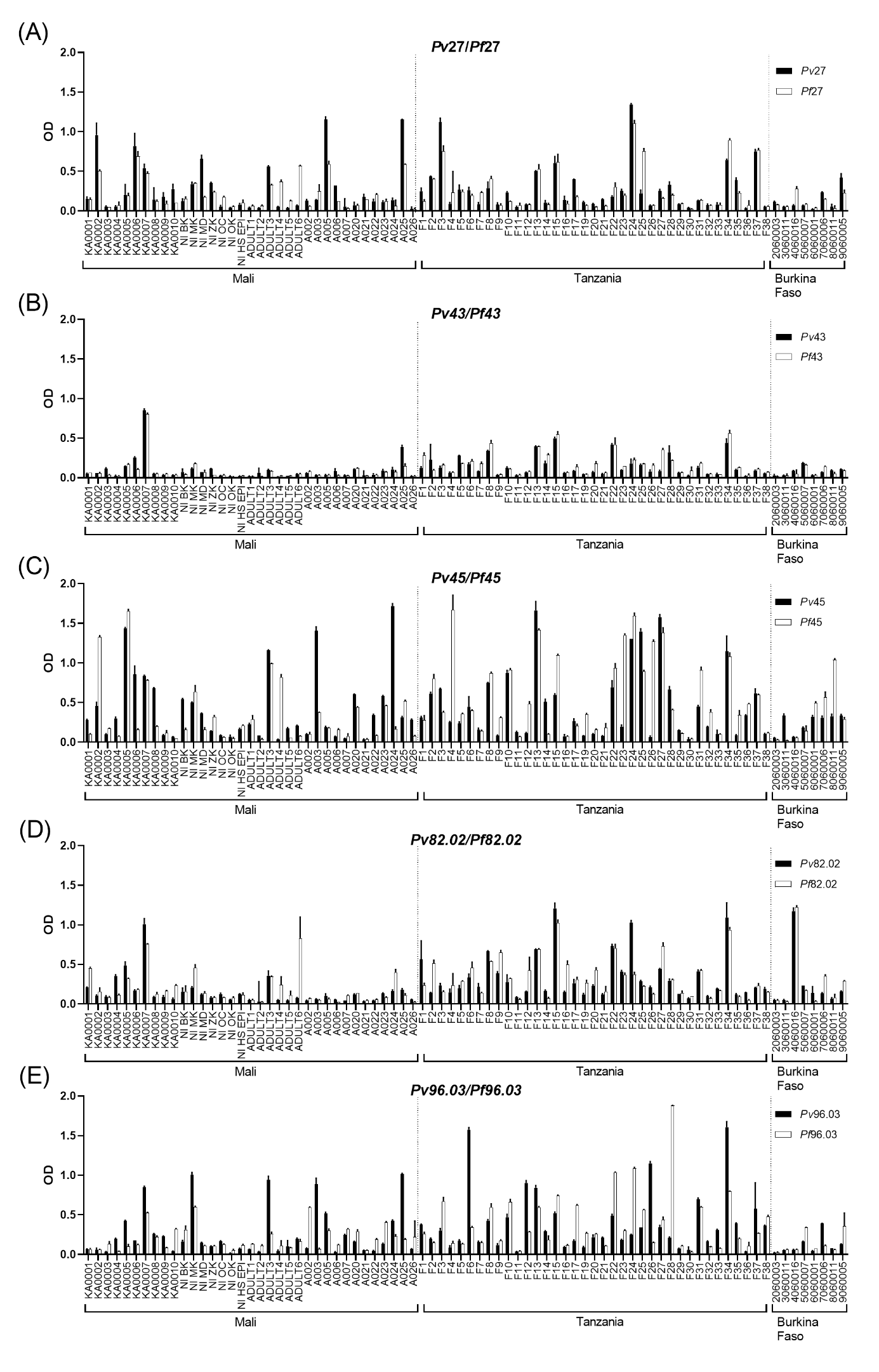

Supplement: Supplementary file 1 [file Data_Sheet_1.DOCX]
